# Improved *Agrobacterium rhizogenes*-mediated hairy root culture system of *Withania somnifera* (L.) Dunal using sonication and heat treatment

**DOI:** 10.1007/s13205-015-0297-2

**Published:** 2015-03-26

**Authors:** Chandrasekaran Thilip, Chellappan Soundar Raju, Kandhan Varutharaju, Abubakker Aslam, Appakan Shajahan

**Affiliations:** Plant Molecular Biology Laboratory, Department of Botany, Jamal Mohamed College, Tiruchirappalli, 620 020 Tamil Nadu India

**Keywords:** *Agrobacterium rhizogenes*, *Withania somnifera*, Hairy roots, Withanolides, Sonication, Heat treatment

## Abstract

An improved *Agrobacterium rhizogenes*-mediated genetic transformation of *Withania somnifera* (L.) Dunal was developed using the bacterial strain R1000 with leaf segment explants of in vitro raised plantlets. Out of the three strains used (R1000, MTCC 2364 and MTCC 532), the strain R1000 proved to be more efficient than others. Among the different conditions tested, the highest (93.3 %) transformation rate was observed after 3 weeks when the explants were subjected to sonication (15 s) and heat treatment (41 °C for 5 min). Transgenic status of the hairy roots was confirmed by PCR using *rol* B-specific primers. HPLC analysis showed the ability of hairy roots to synthesize withaferin A and withanolide A, both steroidal lactones of medicinal value. This protocol offers new avenue in *A. rhizogenes*-mediated hairy root induction and is useful for large-scale production of these bioactive compounds from *W. somnifera*.

## Introduction

*Withania somnifera* (L.) Dunal (Solanaceae) is an important medicinal plant which yields pharmaceutically active compounds called withanolides (Praveen and Murthy 2012). They are chemically named as 22-hydroxy ergostane-26-oic acid 26, 22-δ-lactones which are c-22-steroidel lactones (Sabir et al. 2011). Roots of *W. somnifera* contain major withanolides like withaferin A and withanolide A and they possess specific therapeutic properties. Withaferin A is recognized for its anticancer properties and is reported to inhibit cell growth of various human cancer cell lines including lung cancer—NCl-H46 (Choudhary et al. 2013). Withanolide A has recently been credited with a neotropic agent for recovery from nervous degeneration dendrites formation and its branching, and hence it can be a promising compound for the treatment of neural degeneration types of disease like Parkinson’s and Alzheimer’s disease (Kuboyama et al. 2005; Sabir et al. 2011). Other biological activities of *W. somnifera* include cholinesterase inhibition (Choudhary et al. 2004, 2005), anti-inflammatory via COX-2 enzyme inhibition (Jayaprakasam and Nair 2003), antibacterial activity and sex hormone deficiency regulation (Kiasalari et al. 2009), antiglycation (Maurya et al. 2008), and antipyretic activities (Ali et al. 1997).

Since the roots of *W. somnifera* contain a number of therapeutically applicable withanolides, mass cultivation of roots in vitro will be an efficient technique for the large-scale production of this secondary metabolite. The development involves root culture system that would offer unique opportunity for producing root drug in the laboratory without having to depend on field condition. Such in vitro production of secondary metabolites helps to evade the problems related to genetic and epigenetic variations found in field-grown plants (Praveen and Murthy 2010). Further, constituents of withanolides have differed with the variety of tissue type and growth conditions in field (Sangwan et al. 2007). This causes difficulties in the compositional standardization of herbal formulations and the commercial exploitation of this plant (Chatterjee et al. 2010).

*A. rhizogenes*-mediated hairy root production is an important tool for biosynthesis of valuable secondary metabolites like withanolides. Hairy roots are considered a very good system for continuous synthesis of valuable metabolic compounds in an aseptic condition in the absence of expensive growth regulators in the culture medium (Giri and Narasu 2000; Hu and Du 2006). For this reason, *A. rhizogenes*-mediated hairy root culture has been utilized in several pharmaceutically important plants for the production of secondary metabolites (Le Flem-Bonhomme et al. 2004; Zhao et al. 2004; Fu et al. 2005). However, *A. rhizogenes*-mediated hairy root induction in *W. somnifera* is limited due to the lack of available and efficient hairy root induction procedure (Murthy et al. 2008; Sivanandhan et al. 2013). Alternative approaches for an efficient hairy root induction are valuable for large-scale production of withanolides.

Various efforts have been made to overcome problems associated with host/tissue to increase the number of infection sites, such as use of supervirulent *Agrobacgerium* strains and addition of some compounds to the co-cultivation medium. Recently, sonication-assisted *Agrobacterium*-mediated transformation (SAAT) attracts much attention in several plant species (Liu et al. 2005; Subramanyam et al. 2011). It has been successfully applied for hairy root production in *Papaver somniferum* (Le Flem-Bonhomme et al. 2004) and *Verbascum xanthophoeniceum* (Georgiev et al. 2011). It holds great promise for the enhancement of hairy root production. The advantage of this method is that the cavitations caused by sonication cause thousands of microwounds on the surface of the explants. These microwounds permit *Agrobacterium* to penetrate deeper and more completely throughout the explant than conventional wounding, increasing the probability of infection to host cells (Liu et al. 2005). Further, transformation efficiency in *Agrobacterium* was also improved in several plant species by the use of heat treatment (Khanna et al. 2004; Hiei et al. 2006; Gurel et al. 2009).

The present study was conducted with the major objective of improving the transformation efficiency of *A. rhizogenes* for hairy root production in *W. somnifera* by means of sonication and heat treatments. This system provides an efficient tool for attaining better transformation efficiency and is useful for commercial in vitro production of withanolides.

## Materials and methods

### *A. rhizogenes* strains and hairy root induction

*A. rhizogenes* strains (R1000, MTCC 2364 and MTCC 532) stored at −80 °C were used for hairy root induction (Saravanakumar et al. 2012). In order to induce hairy roots, overnight YEP (Yeast extracts peptone) liquid medium grown (80 rpm at 28 °C for 16 h; OD_600nm_  = 1.0) bacterial suspensions were centrifuged at 8000 rpm for 10 min. For co-cultivation, leaves of 1-month-old in vitro grown plantlets of elite *W. somnifera* accession (ACCN 06) were cut along with midrib into small segments (~0.5 cm × 0.5 cm) and were transferred in Murashige and Skoog (1962) liquid medium containing bacterial suspension and 100 µM acetosyringone for 30 min (Aslam et al. 2010; Mohamed Rafi et al. 2010). The pH of the medium was adjusted to 5.7 ± 0.1 before autoclaving at 121 °C and 104 K Pa for 15 min. After removing the excessive bacteria culture with MS liquid medium and dried on sterile filter paper (Whatman No. 1), the transformed explants were placed on MS solid medium [0.8 % agar (w/v); Sigma–Aldrich] containing petri dishes for 2 days at 25 ± 2 °C in darkness. Two days after co-cultivation, explants were placed on MS basal medium supplemented with 200 mg l^−1^ cefotaxime (Duchefa Biochemie, Netherlands). Further, these explants were subcultured on the same medium at 2 days intervals and the hairy root-induced explants were counted for the analysis of transformation efficiency. Transformation efficiency (%) was calculated as total number of explants which induced hairy roots/total number of explants cultured × 100.

### Sonication and heat treatment on hairy root induction

In order to analyze the influence of heat treatment on transformation efficiency of *A. rhizogenes*, explants were immersed in MS liquid medium containing strain R1000 suspension with 100 µM acetosyringone and they were incubated on different temperatures of 39, 41, or 43 °C for 3, 5, 7, or 10 min. After heat treatment, explants were incubated at 25 ± 2 °C for 20 min and co-cultivated on MS solid medium for 2 days. Then these explants were cultured on hairy root induction medium 200 mg l^−1^ cefotaxime to induce hairy roots.

For sonication, the tubes containing leaf explants in the abovesaid condition were placed on sonicator (Ultrasonic bath sonicator, PCI Analytics Pvt Ltd^®^, Mumbai, India) for 5, 10, 15, or 20 s sonication followed by heat treatment at 41 °C for 5 min. Then they were incubated 25 ± 2 °C for 20 min. The infected explants were further co-cultivated on MS solid medium for 2 days. The co-cultivated explants were inoculated on MS solid medium amended with cefotaxime (200 mg l^−1^) and subcultured at an interval of 2 days to induce hairy roots. The efficiency of transformation was recorded after 3 weeks.

### PCR analysis of transgenic roots

The Polymerase chain reaction (PCR) was used to detect the Ri T-DNA integration in the plant genome (Eppendorf Mastercycler^®^, Germany). Genomic DNA from transformed hairy roots as well as non-transformed roots (normal root from in vitro plants as negative control) was extracted by CTAB method as mentioned by Saravanakumar et al. (2012). Plasmid DNA from *A. rhizogenes* was isolated as described by Bulgakov et al. (1998) and used as positive control. PCR amplification was performed to detect the 423 bp *rol* B gene fragment using specific primer set: forward primer 5′ GCTCTTGCAGTGCTAGATTT 3′ and reverse primer 5′ GAAGGTGCAAGCTACCTCTC 3′. The amplification reaction volume was 25 μl using PCR buffer containing 1.5 mM MgCl_2_, 200 μM of each dNTPs, 200 pM each of primers, 100 ng genomic DNA, and 2 U *Taq* polymerase. The PCR conditions were 94 °C for 5 min (initial denaturation), 42 cycles of 94 °C for 1 min, 52 °C for 1.5 min and 72 °C for 2 min, and a final extension at 72 °C for 10 min. Amplified products were detected by ultraviolet light after electrophoresis in 1.2 % agarose gel stained with ethidium bromide.

### Establishment of hairy roots and HPLC analysis

Hairy roots of 3 weeks culture were subcultured into hormone-free MS liquid medium. The cultures were maintained at 90 rpm in an orbital shaker (LM-570RD, YIHDER^®^ Thiwan) under 25 ± 2 °C in 24 h darkness. Hairy roots were subcultured to fresh media every 7 days of culture.

Dried hairy roots were ground into fine powder (1 g DW) extracted with methanol (10 ml) under sonication for 30 min. HPLC analysis of withanolides was performed according to a modified method of Sivanandhan et al. (2012). The analytical HPLC experiments were performed with High-Performance Liquid Chromatography (LC 2006 System, Japan^®^) equipped with variable wavelength detector operating at 250 nm. Separations were carried out with reverse-phase ODSSUPELCOSILTM column C18 (25 cm × 4.6 mm, 5 µm particle) with methanol:water (65:35) as an eluent at a flow rate of 1 ml min^−1^. Withaferin A and withanolide A standards were procured from Natural Remedies (Bengaluru, Karnataka, India).

### Statistical analysis

Each treatment comprised 25 explants in replicates of three. The number of explants responded, the number of hairy roots, and percentage of transformation efficiency were recorded after 3 weeks of culture. All data were subjected to one-way ANOVA followed by the statistical significance test. The significant difference among the mean ± standard error (SE) was carried out using Duncan’s Multiple range test and significance level of *P* < 0.05 (IBM, SPSS ver. 19).

## Results and discussion

### Effect of *A. rhizogenes* strains on hairy root induction

**Fig. 1:**
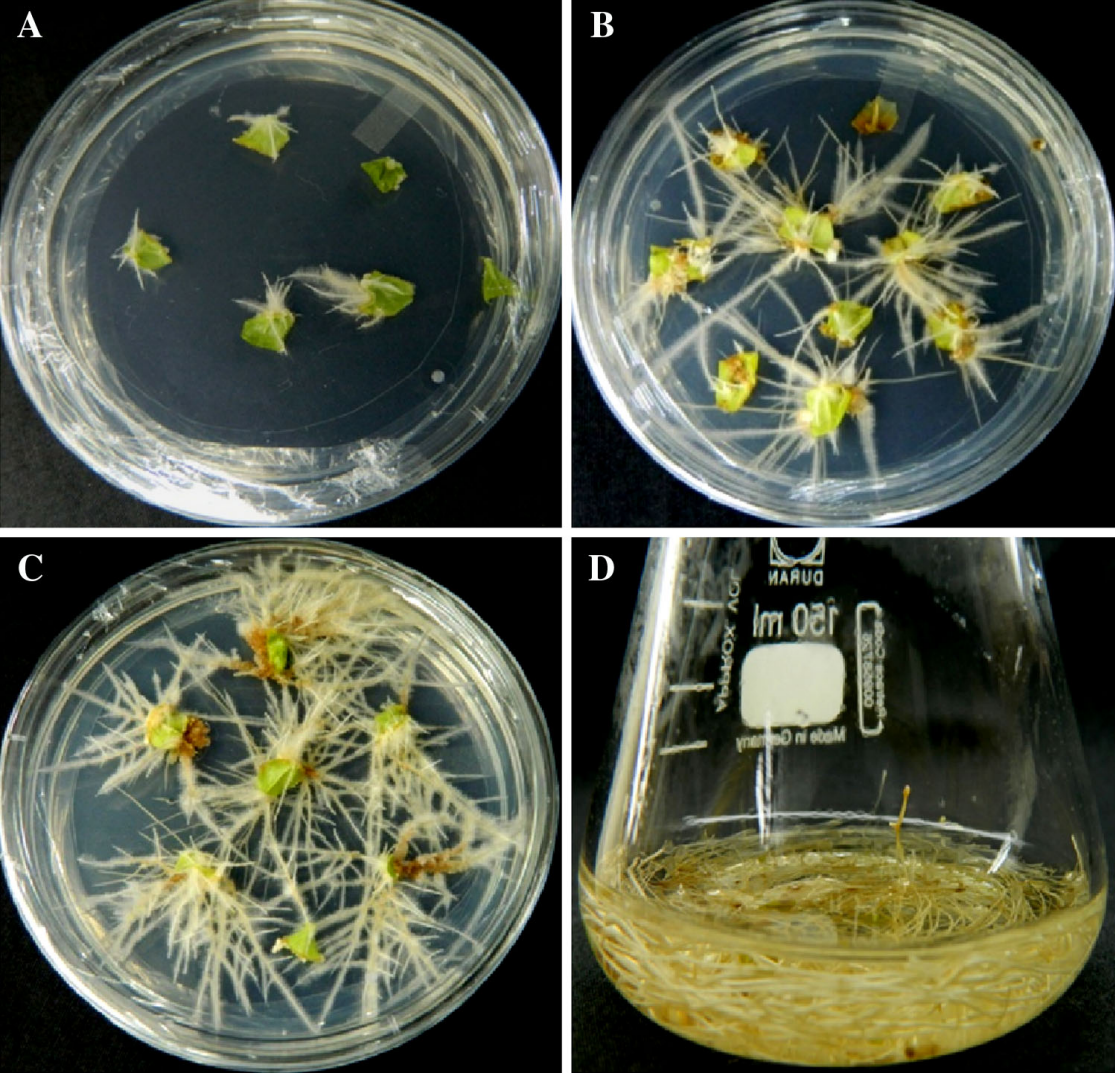
Hairy root culture of *W. somnifera*. **a** Hairy root initiation from leaf explant after co-cultivation with *A. rhizogenes* strain R1000 (after 7 days). **b** Hairy root proliferation after 3 weeks in heat-treated culture (41 °C for 5 min). **c** Hairy root proliferation after 3 weeks in sonication (15 s) and heat treatment (41 °C for 5 min). **d** Proliferative mass accumulation of hairy roots cultured in hormone-free MS basal liquid medium (after 4 weeks of culture)

**Fig. 2:**
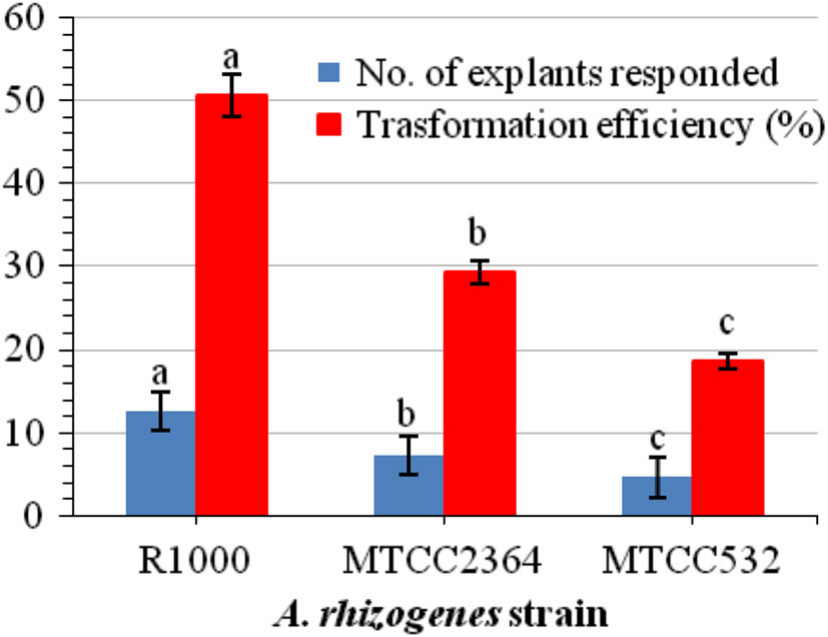
Efficiency of *A. rhizogenes* strain on hairy root induction. Data represented mean ± SE, of the three independent experiments. Mean values showed by different letters are significantly different at *P* < 0.05 (DMRT)

*A. rhizogenes* strains R1000, MTCC 2364, and MTCC 532 were used to determine the transformation efficiency toward hairy root induction in *W. somnifera*. These three strains showed different levels of infection and there were no hairy roots from control explants. *A rhizogenes* strain R1000 was found to be more effective for inducing hairy roots and showed maximum 50.6 % transformation efficiency (Figs. 1a, 2). MTCC 2364 showed less efficiency (29.3 %) than R1000, and MTCC 532 was the least efficient (18.6 %). Influence of *A. rhizogenes* strain on hairy root induction frequency has been documented earlier in several plant species (Sujatha et al. 2013). R1000 strain was widely used for hairy root induction in many plants such as *Torenia foarnieri* (Tao and Li 2006), *Gentiana macrophylla* (Tiwari et al. 2007), and *Artemisia vulgaris* (Sujatha et al. 2013). The infection process is highly dependent on successful transfer of Ri T-DNA into the host cells (Mariashibu et al. 2013). This intrinsic capacity of Ri plasmid must be higher in R1000 when compared to other strains as inferred by other reports also (Wang and Fang 1998).

### Effect of heat treatment on hairy root induction

**Fig. 3:**
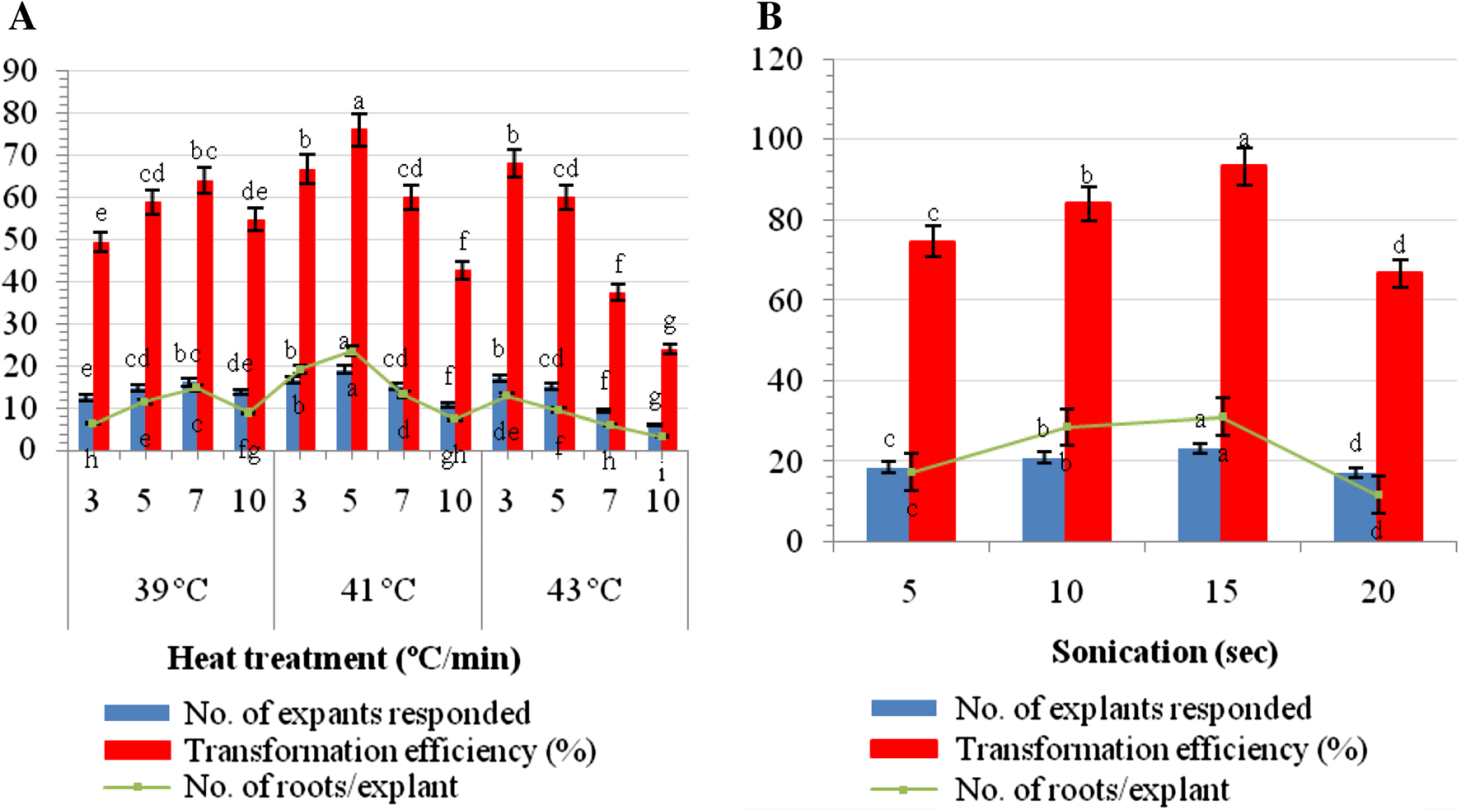
**a** Influence of heat treatment on transformation efficiency and **b** sonication on hairy root induction. Data represented mean ± SE, of the three independent experiments. Mean values showed by different letters are significantly different at *P* < 0.05 (DMRT)

Heat treatment is a common approach in tissue culture and *Agrobacterium*-mediated transformation studies in several plant species (Hiei et al. 2006). The significant enhancement of *Agrobactreium* transformation efficiency using heat treatment was achieved in banana (Khanna et al. 2004) and sorghum (Gurel et al. 2009). In the present study, we analyzed for the first time to evaluate the effect of heat treatment toward the transformation efficiency of *A. rhizogenes* in *W. somnifera*. The explants were infected with *A. rhizogenes* at various temperature of 39, 41, 43, or 45 °C and duration of 3, 5, 7, or 10 min. Our preliminary results showed that 41 °C heat treatment for 5 min was the most effective for higher percentage (76.0 %) of hairy root induction (Figs. 1b, 3a). When heat treatment at 41 °C was extended from 5 to 7 or 10 min, there was a drastic reduction in the hairy root induction frequency.

### Effect of sonication and heat treatment on hairy root induction

Sonication-assisted *Agrobacterium*-mediated transformation is an improved approach to enhance the efficiency of *Agrobacterium*-mediated transformation of many low or previously known to be non-susceptible plant species (Trick and Finner 1997, 1998; Subramanyam et al. 2011). The enhanced transformation efficiency using SAAT probably results from thousands of microwounds on and below the surface of the target tissue caused by sonication. These microwounds facilitate *Agrobacterium* to penetrate deeper into the target tissue and provide efficient delivery of T-DNA for transformation (Liu et al. 2005; Subramanyam et al. 2011). Subramanyam et al. (2011) and Mariashibu et al. (2013) successfully obtained transformed plants by sonicating target tissues with *A. tumifaciens* cultures. No reports on transformation with SAAT method are available for hairy root induction in *W. somnifera*. We investigated the influence of sonication on transformation efficiency of *A. rhizogenes* in *W. somnifera* by sonication of explants in suspension for different time duration of 5, 10, 15, or 20 s followed by heat treatment of 41 °C for 5 min. Among the different time durations, a 15 s sonication proved to be the best and produced 93.3 % of transformation efficiency after 3 weeks (Figs. 1c, 3b). Beyond 5, 10, or 20 s, percentage of transformation efficiency was reduced. This might be because of excess tissue damage due to prolonged sonication.

*A. rhizogenes*-mediated hairy root induction has been documented earlier in *W. somnifera* by several authors (Ray et al. 1996; Kumar et al. 2005; Sivanandhan et al. 2014). Ray et al. (1996) reported that the withanolide D productivity was higher in (0.181 mg l^−1^ day^−1^) transformed roots than in untransformed roots (0.026 mg l^−1^ day^−1^). Kumar et al. (2005) observed the antioxidant activity in *A. rhizogenes* transformed hairy roots. Both of them failed to analyze the hairy root induction frequencies in *W. somnifera*. Recently, Sivanandhan et al. (2014) observed that the leaf explants of *W. somnifera* showed higher (88.4 %) frequency of hairy root induction than in cotyledon explants (64.2 %) when co-cultivated with *A. rhizogenes* strain R1000. The present study reports that the highest transformation efficiency (93.3 %) was obtained when the leaf explants of *W. somnifera* were subjected to sonication and heat treatment during transformation with *A. rhizogenes*. This protocol can be used for hairy root production on a large scale at a faster rate compared to previous methods of hairy root induction in *W. somnifera*.

### PCR analysis for confirmation of transgenic status

**Fig. 4:**
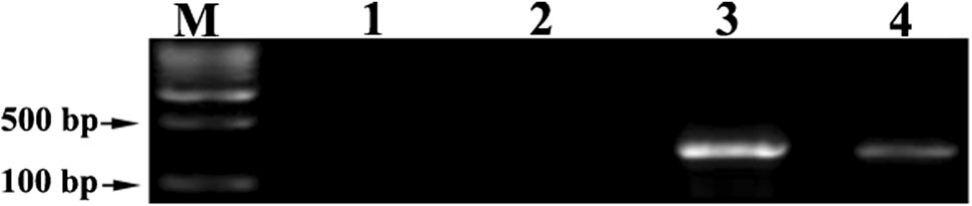
PCR detection of *rol* B gene in hairy roots. *Lane M* molecular weight marker (1000 bp); *lane 1*, water control; *lane 2*, negative control (non-transformed roots); *lane 3*, *rol* B positive control (Ri plasmid); *lane 4*, *rol* B transformed hairy roots

In the present study, to confirm the integration of T-DNA from the *A. rhizogenes* into the hairy roots, *rol* B gene was tested using *rol* B-specific primers (Fig. 4). *A. rhizogenes* transfers two independent T-DNAs (T_L_-DNA and T_R_-DNA) to the host plant genome (Nilsson and Olsson 1997). T_L_-DNA to be the most important virulent factor and it is essential to induce hairy roots (Sujatha et al. 2013). The sequence analysis of T_L_-DNA identified 18 open reading frames (ORF), four of which are essential for hairy root induction namely ORF 10 (*rol* A), ORF 11 (*rol* B), ORF 12 (*rol* C), and ORF (*rol* D) (Georgiev et al. 2011). White et al. (1985) identified that *rol* B has a central role in pathogenecity. In this study, DNA from agropine type of *A. rhizogenes* strain R1000 served as the positive control and normal roots of in vitro plants served as the negative control. PCR results showed that all the hairy root lines contained 423 bp *rol* B gene fragment, which were the parts of T-DNA of Ri plasmid (Fig. 4).

### Establishment of hairy root culture and HPLC analysis

**Fig. 5:**
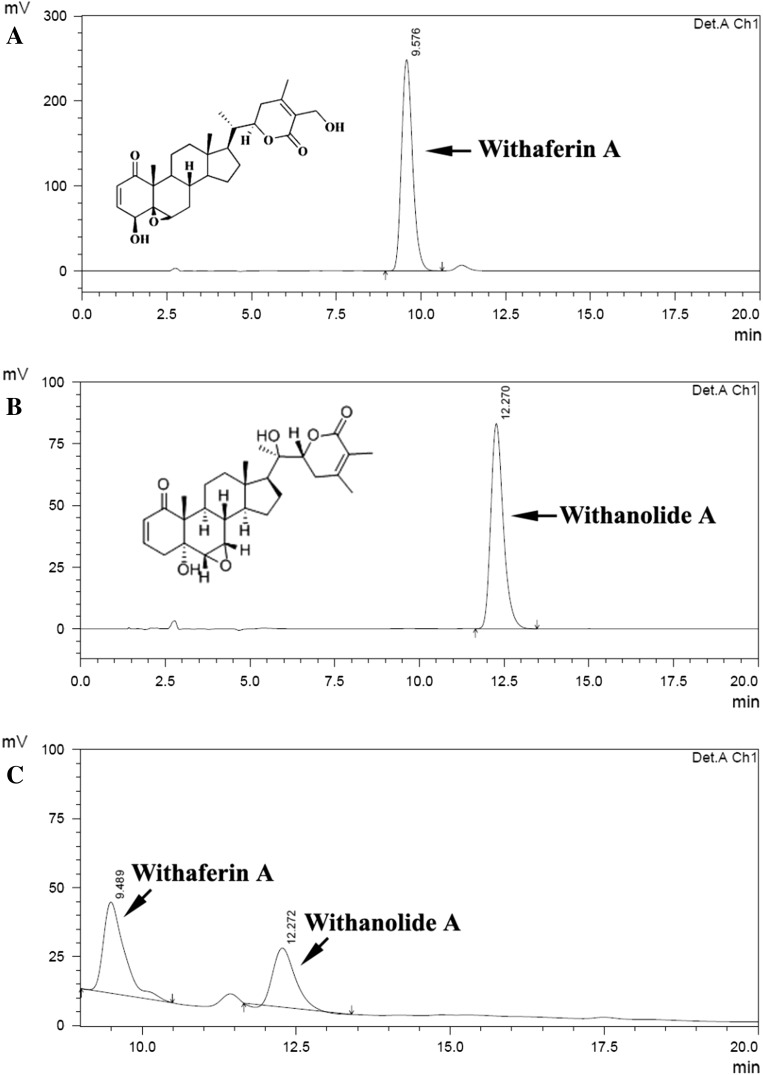
HPLC analysis of withanolide production in hairy root culture. **a** Standard withaferin A, **b** standard withanolide A and **c** Withanolides (withaferin A and withanolide A) from sonication (15 s) and heat treatment (41 °C for 5 min)-assisted hairy roots culture

The potential growth of hairy roots in liquid (MS) shake-flask culture without hormone was successfully achieved in the present study (Fig. 1d). The HPLC profile of withaferin A and withanolide A from the hairy root of *W. somnifera* is depicted in Fig. 5a–c. The results confirmed a considerable amount of withaferin A (6.17 mg g l^−1^ DW) and withanolide A (3.82 mg g l^−1^ DW) in the hairy roots, which were identified with peaks corresponding to standard withanolide markers. The accumulation of higher levels of withanolides in hairy roots could be due to the effect of T-DNA integration on the secondary metabolite production as reported in many plant species (Bulgakov 2008; Shkryl et al. 2008; Bansal et al. 2014). Although hairy roots of the Italian *W. somnifera* were induced by *A. rhizogenes*, they did not accumulate withanolides (Vitali et al. 1996). The present study found the presence of withanolides in transformed hairy root culture of the Indian *W. somnifera* produced using *A. rhizogenes*. Similarly, Bandyopadhyay et al. (2007) and Murthy et al. (2008) also reported the presence of withanolides in transformed hairy root cultures of Indian *W. somnifera*.

## Conclusion

The highest percentage of hairy root induction was obtained with *A. rhizogenes* strain R1000 into *W. somnifera* leaf explants through 15 s sonication and heat treatment at 41 °C for 5 min. Furthermore, these transformed hairy roots are able to produce bioactive compounds. To the best of our knowledge, this is the first report of *A. rhizogenes-*mediated transformation using sonication and heat treatment in *W. somnifera*. This system provides an efficient tool for hairy root production and is useful for commercial production of withanolide under in vitro condition.
